# Impact of Artificial Intelligence-supported Triage Systems on Emergency Department Management: A Comparison of Infermedica, Emergency Severity Index, and Manchester Triage System

**DOI:** 10.5811/westjem.48989

**Published:** 2026-02-27

**Authors:** Erkan Boğa

**Affiliations:** Esenyurt Necmi Kadıoğlu State Hospital, Emergency Medicine Service, Istanbul, Türkiye

## Abstract

**Objective:**

The surge in the number of emergency department (ED) visits due to a growing population, aging society, and easier access to healthcare highlights the need for an effective triage process. Our goal in this study was to compare the clinical and operational performance of a triage system supported by artificial intelligence (AI) with two traditional methods—the Emergency Severity Index and the Manchester Triage System—in a high-volume ED.

**Methods:**

In this prospective study, 18,000 adult patients were randomized equally to one of the three triage systems. Primary and secondary outcomes included patient wait time, complication and mortality rates, resource utilization, medical errors, legal issues, and patient satisfaction.

**Results:**

Compared with the Manchester Triage System, the AI-supported system was associated with significantly lower in-ED mortality (OR 0.39, 95% CI, 0.32–0.47; P < .001) and lower complication rates (4.42% vs 10.25%), as well as higher patient satisfaction scores (9.0 vs 7.0; P < .001). Resource utilization was also more balanced in the AI-supported triage cohort.

**Conclusion:**

The AI-assisted triage system showed favorable clinical and operational patterns relative to traditional methods. However, the single-center design and short observation period limit generalizability, and causal inferences could not be firmly established.

## INTRODUCTION

Emergency departments (ED) are among the busiest and most dynamic units of the healthcare system. They are the primary service point where the care and treatment of patients requiring urgent intervention due to sudden illnesses, traumas, and acute health problems are carried out. In recent years, the growing population, aging society, and easier access to healthcare services have led to a significant increase in the number of patients visiting EDs.^1^ This surge in demand makes it challenging for healthcare personnel, working with limited resources and under time pressure, to quickly and accurately assess the urgency of patients, highlighting the necessity of an effective triage process.^2^

Triage is the process of classifying patients based on the severity of their conditions to manage the patient load in EDs and ensure efficient use of resources.^3^ An effective triage system ensures that critically ill patients are quickly identified and prioritized for intervention, while also contributing to the efficient use of resources in managing emergency cases. Although traditional triage systems fulfill this purpose, they are criticized for occasionally being ineffective and for disrupting patient care due to the increasing patient load and the complex structure of EDs.^4^,^5^ It is argued that these systems, operating under limited resources, may fail to assess patients’ conditions quickly and accurately, potentially leading to delays in diagnosing or prioritizing critical patients for intervention.

The development of artificial intelligence (AI)-based triage systems offers a pathway to optimize patient management in the ED and enhance the accuracy of the triage process. These systems have the capability to systematically analyze patient data such as patient symptoms, vital signs, and medical history to support risk assessment. Infermedica (Infermedica, LLC, Wroclaw, Poland) is one of several AI-based triage systems designed to assist clinicians in risk assessment, serving as an example of the integration of AI algorithms into healthcare services in evaluating the urgent need for care based on the patient’s symptoms.^6^,^7^

When a patient presents to the ED, their age, sex, and current symptoms are entered into the Infermedica system. Although the exact number of symptoms required to generate an assessment has not been strictly defined, one to three well-defined symptoms are considered sufficient. These symptoms can be categorized into groups such as pain, fever, or shortness of breath. The system also allows for the inclusion of past medical history and risk factors to be considered during the evaluation. In some implementations, vital signs may also be integrated, depending on how the system is configured.

Infermedica’s AI algorithms analyze the entered symptoms using a comprehensive medical knowledge base to evaluate potential conditions. The system calculates how urgently the patient needs intervention by assessing the severity and nature of the reported symptoms. It then provides a risk score that reflects the patient’s overall condition and urgency level.^8^,^9^ This score helps determine whether the patient requires immediate treatment or it is safe for them to wait for treatment. Based on this assessment, the system advises healthcare personnel which triage category the patient should be placed in.

Infermedica typically “aligns”—meaning it is designed to correspond—with the color-coded triage systems commonly used in EDs (eg, red for emergency, yellow for moderate urgency, green for non-urgent cases). However, this does not mean it is limited to three categories. Infermedica can support more nuanced prioritization levels if the clinical workflow requires it, and its output can be adapted to match the specific triage model used in each healthcare facility.

The Emergency Severity Index (ESI) and the Manchester Triage System are traditional triage methods widely used in EDs, both playing a critical role in managing patient flow in these settings.^10^ The ESI is a five-level scoring system commonly used in the United States, by which the triage nurse categorizes patients based on the urgency of their condition and the number of medical resources anticipated to be needed. It is considered an effective tool for expediting patient prioritization.^11^ Although the ESI supports rapid decision-making mechanisms and contributes to the reduction of mortality and morbidity rates, it may be inadequate in cases involving complex or ambiguous symptoms.^12^

On the other hand, the Manchester Triage System used in Europe classifies patients into five urgency levels using color codes—red (immediate); orange (very urgent); yellow (urgent); green (standard); and blue (non-urgent)—based on their clinical symptoms.^13^ While the system facilitates structured decision-making, it can be time-consuming under high patient volumes due to the detailed decision trees and symptom flowcharts that must be navigated for each presenting complaint. This complexity may delay triage decisions, especially when symptoms are atypical or overlap multiple diagnostic pathways, ultimately limiting the system’s efficiency in the fast-paced emergency setting.^14^

**Population Health Research Capsule**

What do we already know about this issue?

*Demand for emergency care places stress on triage systems*.

What was the research question?

*How would an ED triage system supported by artificial intelligence (AI) perform compared to traditional triage systems in clinical, operational, and safety outcomes?*

What was the major finding of the study?

*The AI-supported triage system was associated with lower mortality rates vs the Manchester Triage System (OR 0.39, 95% CI, 0.32–0.47, P < .001)*.

How does this improve population health?

*Triage supported by AI may reduce delays, errors, complications, and mortality, and may improve safety, resource use, and overall emergency care quality*.

In this study we evaluated the ED performance of Infermedica, the ESI, and the Manchester Triage System through clinical results, patient wait times, and incidence of reported complications, mortality and medical errors, as well as resource consumption, legal issues, risk management, and patient satisfaction. This research provides selection guidance for appropriate triage methods and AI technology implementation in ED clinical workflows. These findings help assess how AI-supported triage systems can enhance patient care while improving ED management.

## METHODS

This prospective, comparative study included 18,000 adult patients who presented between October 10–November 1, 2024 to the ED of Esenyurt Necmi Kadıoğlu State Hospital, a secondary-level, public healthcare facility with a high patient volume, receiving an average of 3,000 ED visits per day and over 1,000,000 visits annually. During this 23-day period, the hospital received approximately 69,000 ED visits according to the electronic health record (EHR). From these visits, all adult patients meeting inclusion criteria were identified through the EHR system.

Under routine ED workflow conditions, patients are registered by the central admissions unit and ED nurses subsequently perform triage assessment using a three-level, color-code triage system (red, yellow, green), which is the standard triage model in Türkiye. For the duration of this study, 18,000 eligible patients were randomly selected (after applying exclusion criteria) via a computer-generated sampling algorithm. These patients were then allocated among the three triage systems, controlling for age and sex distributions. This ensured unbiased selection and prevented over-representation of any clinical subgroup within the ED population. We chose this approach to enhance statistical power and enable direct comparison of outcomes across groups. Nurses in the ED triaged 6,000 patients using Infermedica; 6,000 using the ESI; and 6,000 using the Manchester Triage System. All triage methods were applied independently, and nurses were directed to use the system assigned to them.

Patients who presented with unstable vital signs or required immediate life-saving intervention (“red zone”) were excluded from the study to ensure patient safety and avoid interference with emergent clinical care. We also excluded pregnant patients, individuals with multiple ED visits during the study period, transfers to other facilities after stabilization, and cases requiring psychiatric evaluation. These patients were excluded to prevent confounding effects that could have resulted from different clinical pathways. The ED workflows for psychiatric and pregnant patients differ from standard procedures, while repeat visits and inter-facility transfers could have created biases in outcome tracking.

The data-collection process began at the moment of each patient’s arrival to the ED and continued systematically throughout the study period. Data were collected through direct observations by trained healthcare personnel, as well as through the hospital’s EHR system and patient admission records. Specifically, for the Infermedica system, triage data inputs included the patient’s age, sex, one to three clearly defined symptoms (such as pain, fever, or shortness of breath), past medical history and, when available, vital signs. This information enabled the AI-powered system to generate a risk score and recommend an appropriate triage category. Patients were classified into three risk groups based on their clinical condition: good, moderate, and poor. The categorization system enabled researchers to establish a common framework for evaluating all triage methods.

Standardized definitions based on vital signs and clinical presentation replaced system-specific labels such as “emergent” or “non-urgent.” The standardized approach maintained uniform classification throughout both AI-based and traditional triage systems. This classification was made by considering the patient’s vital signs recorded at admission, reported symptoms, and preliminary diagnoses. Patients in the “good risk” group had vital signs within normal limits, exhibited mild symptoms, and did not require urgent medical intervention. Patients in the “moderate risk” group had moderate symptoms and showed borderline abnormalities in some vital signs. These patients required medical evaluation but were not in a life-threatening condition. Patients in the “poor risk” group had one or more severely impaired vital signs, exhibited life-threatening symptoms, or required immediate medical intervention. This classification was standardized and applied across all three triage methods—Infermedica, ESI, and the Manchester Triage System.

We defined the study’s primary variables as 1) clinical outcomes, which included mortality and the development of complications, both assessed during the patient’s stay in the ED; and 2) outcome measures, primarily patient wait time, which we defined as the interval from the patient’s arrival and ED registration to initial clinical evaluation by a physician. This measure included the triage assessment period but excluded any additional delays caused by bed assignment or transfer processes after the initial evaluation. Treatment duration was calculated from the time of arrival to the point of discharge or the decision to admit the patient to an inpatient unit. The timing data were extracted from the EHR, which automatically timestamped patient arrival and first physician contact. We analyzed these primary variables to compare the effects of the three triage methods on ED operations and patient outcomes.

Secondary outcome measures included clinical complications and in-ED mortality. For the purposes of this study, we defined “recovered” as patients who either were treated and discharged directly from the ED without requiring admission to the intensive care unit (ICU) or patients who died in the ED. (General ward patients who did not need ICU care were not categorized as “recovered” because they were admitted to the hospital for observation.) We defined mortality as death occurring during the ED visit, based on discharge or death records in the hospital information system. Long-term mortality (eg, 30-day or post-discharge outcomes) was not included in the analysis. Additional secondary outcome measures included resource utilization, patient satisfaction scores, and medicolegal issues together with medical error rates.

We defined resource utilization as the total use of medications, procedures, medical supplies, and staff time during the ED stay. For the measurement of resource use, we obtained data from hospital accounting records and billing data, which included all procedures, materials, and treatments performed in the ED. Through EHR review we obtained the costs of medications and equipment used during the triage process. Additionally, we conducted a total cost analysis by combining the costs of procedures, medications, materials, and personnel to calculate the average costs for patients triaged according to each system. In this way, we determined the financial impacts of ED triage management strategies on resource use.

We measured patient satisfaction using a standardized 10-point Likert-scale survey (1 = not satisfied at all, 10 = very satisfied), and analysis was conducted using average scores. The response rate was 71.1%, with 12,800 of 18,000 patients completing the survey after discharge. The survey included questions assessing satisfaction with wait time, clarity of communication by staff, and perceived fairness of prioritization. Satisfaction scores were analyzed as continuous variables and compared between triage systems. The mode of survey administration (face-to-face or phone) may have contributed to the high response rate, although this effect was not formally analyzed. This methodology ensured that satisfaction measurements were both representative and systematically collected.

### Assessment of Medicolegal Issues

We assessed medicolegal issues associated with triage practices through hospital legal records and the institutional risk-management database. We defined medical error as incorrect triage categorizations that led to delayed or inappropriate treatment choices. Medical error analysis was based on a structured chart review of a representative sample rather than all enrolled patients. A systematic chart review process was established to maintain both objectivity and consistency in error identification. Two board-certified emergency physicians with expertise in clinical audits and error detection performed a structured chart review to identify such errors. The review process evaluated clinical documentation together with treatment timelines and the match between assigned triage categories and subsequent diagnosis or interventions. They reviewed a randomly selected sample of 300 cases from each triage system cohort (900 total) to evaluate triage-related medical errors.

These cases were used for error rate calculation, and do not represent the full 18,000-patient cohort. The reviewers evaluated the appropriateness of triage categorization through assessment of clinical presentation and outcomes. A third senior reviewer intervened to achieve consensus when reviewers disagreed about a case. Inter-rater reliability reached 0.81 according to the Cohen kappa statistic, which indicates strong agreement.

We compared the rates of medical errors across the three triage systems using multivariable logistic regression analysis, controlling for patient age, sex, triage category, initial vital signs, and comorbidities. While we acknowledge that multiple factors may influence patient outcomes in the ED, this analytical model was designed to isolate the independent impact of the triage method on error rates as rigorously as possible.

Each of the three triage methods was applied strictly in accordance with its protocol during the clinical evaluation of the study population. We performed data analysis using SPSS 28.0 statistical software (SPSS Statistics, IBM Corp, Armonk, NY). The normality of data distributions was assessed using the Kolmogorov-Smirnov test. We compared continuous variables using independent sample *t*-tests, and categorical variables with chi-square tests.

While the clinical data covered a three-week period (October 10–November 1, 2024), follow-up for legal claims and complaints extended to six months post-visit, which is a commonly observed window for the filing of preliminary complaints or malpractice claims in Türkiye. Although civil litigation may occur beyond this period, the six-month timeframe allowed for identification of immediate or early legal repercussions, such as formal complaints, internal investigations, and legal consultations initiated by patients or families.

### Nurse Training for Each Triage System

This study entailed a comprehensive training program to ensure that nurses accurately implemented the triage system assigned to them. The 24 hours of training were delivered over three days. Nurses received structured and equal training for all three triage systems. The theoretical component introduced the fundamental principles, decision-making algorithms, and clinical application protocols for each system. The simulation phase included 30 case-based scenarios (10 per system) and 15 high-fidelity simulations designed to enhance nurses’ ability to assess complex and high-pressure patient situations. The practical training involved five supervised sessions per nurse for each triage system. During these sessions, nurses applied the triage protocols to 10 real patients per system, under the direct observation of expert trainers. Their performance was assessed using standardized evaluation rubrics.

To become certified in each triage system, nurses were required to score at least 80% on a standardized theoretical exam and achieve a minimum of 90% accuracy in scenario-based and live clinical assessments. Certification was granted separately for each system and was based on objective performance criteria. To support the training process, detailed educational materials and guidelines tailored to each triage system were provided to the participating nurses. These materials included structured scenario libraries, decision algorithms, and expected clinical responses for each system to ensure nurses were adequately prepared to manage a wide range of patient conditions. Following the training program, nurses’ performance was evaluated through a standardized written test consisting of 30 multiple-choice questions and 15 case-based analysis scenarios, equally distributed across the three triage systems.

Infermedica is an AI-powered decision-support tool that analyzes patient information to assist triage nurses in classifying emergency cases. At the start of triage, the patient’s age, sex, and one to three primary symptoms are entered into the system; additional medical history, risk factors, and vital signs can also be included. The system uses a structured and adaptive question flow, dynamically refining its queries to clarify symptom severity and clinical context. Its underlying algorithm references a large, curated medical knowledge base to generate a risk score and recommend an urgency category aligned with standard triage color codes (red for immediate, yellow for urgent, green for non-urgent).

Real-time data processing and automated risk-stratification may help reduce delays caused by manual decision-making and extensive flowcharts, while standardized scoring can decrease inter-observer variability and potential errors. Additionally, the system’s broad medical database supports recognition of atypical or overlapping symptoms. These potential benefits should be interpreted with caution because real-world performance depends on training, workflow integration, and input quality. Further multicenter research is needed to confirm these observations.

Regular feedback was delivered weekly during the two-week post-training period through follow-up meetings and digital communication platforms. This feedback was designed to reinforce protocol adherence and address individual knowledge gaps. In addition, post-training support included the assignment of clinical supervisors, available on site during each shift, to answer questions and assist nurses as they applied triage systems in real-time patient settings. This structured mentoring was designed to promote consistency, minimize variance in protocol implementation, and reduce the risk of medical errors during the data collection phase.

We analyzed and compared these parameters across the three triage methods using independent sample *t*-tests and logistic regression analyses. All models were adjusted for potential confounding variables, including age, sex, triage category, initial vital signs (heart rate, blood pressure, respiratory rate, oxygen saturation, temperature), and past medical history (eg, diabetes, cardiovascular disease, immunosuppression). Mean durations, odds ratios (OR), and 95% confidence intervals were calculated, and statistical significance was set at *P* < .05. Through these measurements, we evaluated the differences between Infermedica, the ESI, and the Manchester Triage System for their effects on clinical outcomes and risk management.

We used multivariable logistic regression models to determine the independent effects of the three triage method on binary outcomes, including complication rates, in-ED mortality, high resource utilization, and occurrence of legal issues. These models were adjusted for confounding variables such as age, sex, triage level, initial vital signs (heart rate, blood pressure, respiratory rate, temperature, SpO_2_), and comorbidities (eg, diabetes, cardiovascular disease, immunosuppression). Odds ratios (OR) and 95% confidence intervals were calculated. A p-value of < .05 was considered statistically significant for all analyses. No formal correction for multiple comparisons (eg, Bonferroni) was applied; therefore, the statistical findings should be interpreted with caution due to the increased risk of type I error.

A structured chart review was conducted using the methodology described by Worster and Bledsoe (2005). Two independent emergency physicians reviewed a random sample of 300 cases from each triage group. Inter-rater reliability was assessed using the Cohen kappa coefficient, which was calculated at 0.81, indicating strong agreement. Legal issues were identified through follow-up of institutional risk-management records and hospital legal databases for a six-month period after each patient’s ED visit—an appropriate timeframe in the Turkish healthcare system for the reporting and filing of complaints or legal actions.

This study evaluated short-term mortality, defined as death occurring within the ED visit among patients who presented during the one-month study period. Mortality data were extracted directly from the EHR and confirmed by discharge or death summaries. Long-term mortality (ie, 30-day or post-discharge outcomes) was not within the scope of this study.

All patient data were anonymized and handled in accordance with standard data protection protocols. Access was limited to authorized research team members. We conducted the study independently, with no financial or professional ties to the companies or developers of the evaluated triage systems.

### Ethics Approval

This study was approved by the Non-Interventional Clinical Research Ethics Committee of Istanbul Medipol University (Approval No: 289; June 3, 2025). The research was conducted in accordance with the ethical principles stated in the Declaration of Helsinki. Because the study involved analysis of routinely collected anonymized ED data and no direct interventions beyond standard care, the requirement for individual informed consent was waived by the ethics committee. Approval for this study was obtained from the Non-Interventional Clinical Research Ethics Committee of Istanbul Medipol University (Approval No: 289; June 3, 2025). We cobducted all procedures in accordance with the Declaration of Helsinki; the ethics committee waived the requirement for individual informed consent due to the use of anonymized clinical data.

## RESULTS

**Figure 1:**
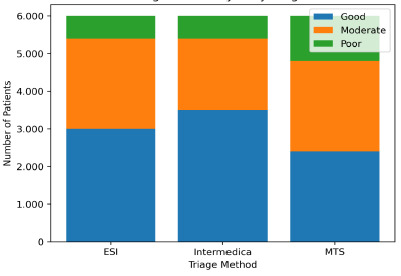
Risk category distribution by triage method. *ESI*, Emergency Severity Index; *MTS*, Manchester Triage System.

Demographic characteristics were balanced across the three triage cohorts, ensuring comparability (Table 1). The categorization of patients into “good,” “moderate,” and “poor” risk groups was based on predefined clinical criteria, as described in the *Methods* section. These definitions consider clinical presentation, vital signs, and initial triage assessment parameters (Figure 1, Table 2).

The risk-stratification system of Infermedica placed most patients in the “good risk” group, which corresponded to lower complication and mortality rates (Tables X–Y). The risk-stratification system of Infermedica demonstrated better clinical severity assessment, which led to safer patient care practices.

**Figure 2:**
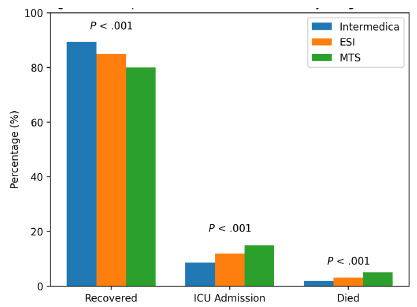
Comparison across three triage cohorts of the percentages of patients who recovered, were admitted to the intensive care unit, or died. *ESI*, Emergency Severity Index; *ICU*, intensive care unit; *MTS*, Manchester Triage System.

Clinical outcomes of the three triage methods are presented in Figure 2. As shown in the table, Infermedica had the highest proportion of patients categorized as “recovered,” while the Manchester Triage System showed the highest ICU transfer and mortality rates.

The results indicate that patients triaged using the Infermedica method had the highest recovery rate, along with the lowest rates of ICU transfer and in-ED mortality. Infermedica yielded the highest recovery and lowest ICU admission and mortality rates among all systems, indicating favorable clinical outcomes. While these findings suggest that Infermedica may be associated with improved clinical outcomes, it is important to interpret these results with caution. The observed differences cannot be solely attributed to the triage method itself, and further investigation is needed to determine causality. This limitation is acknowledged in the *Discussion* section.

The observed differences in risk-group distribution could indicate different levels of triage accuracy, but it is important to consider the possibility of residual imbalance even after stratified randomization. Therefore, causality should be inferred without caution.

Patient satisfaction outcomes for all three triage methods are summarized in Table 3. The AI-supported Infermedica triage achieved the highest average satisfaction score, indicating improved patient experience. The ESI method achieved moderate satisfaction, while the Manchester Triage System showed the lowest performance in terms of patient satisfaction.

**Figure 3:**
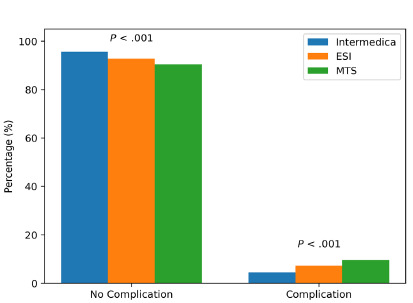
Complication rates by triage method. *ESI*, Emergency Severity Index, *MTS*, Manchester Triage System.

Overall, Infermedica triage was associated with the lowest complication rate followed by ESI, while the Manchester Triage System showed the highest complication rate. Of 6,000 patients triaged with Infermedica, 265 experienced complications (4.42%), compared to 429 patients (7.15%) triaged using the ESI and 615 patients (10.25%) with the Manchester Triage System. Complication outcomes for all triage methods are summarized in Figure 3.

Complication rates were lowest with Infermedica, further supporting its safety profile. The Manchester Triage System had the highest complication rate and was, therefore, riskier. The results consistently show differences across the three triage methods; Infermedica was associated with more favorable values in several metrics, although causality cannot be inferred (eg, clinical outcomes, patient satisfaction, error rates), and these findings should be interpreted with caution. The observational study design prevents drawing causal relationships from the data because observed differences could stem from staff adaptation and patient flow patterns and institutional excitement about the new AI system during its initial deployment. The balanced randomization method did not eliminate all operational variance, which could have impacted real-time triage accuracy.

**Figure 4:**
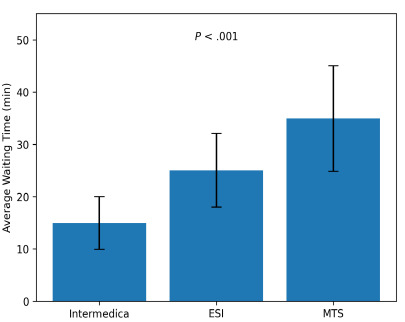
Average patient wait times by triage method (mean ± SD) with findings reflecting how triage systems affect emergency department flow. *ESI*, Emergency Severity Index; *MTS*, Manchester Triage System; *min*, minutes.

The patients who received Infermedica triage spent less time waiting on average than patients who were triaged using the ESI or the Manchester Triage System. The observational nature of the study prevents us from establishing causality. Average patientwait times are shown in Table 4, and Figure 4 demonstrates how wait times impact ED workflow. While the statistical models controlled for important confounding variables they lacked a formal multiple comparison adjustment, which increases the chance of type I errors. The reported *P*-values should be considered exploratory rather than confirmatory because no formal correction for multiple comparisons was applied. In terms of patient wait times, Infermedica showed the shortest average and median durations, followed by ESI, while the Manchester Triage System was associated with the longest wait times.

Emergency department resource utilization across the three triage methods is summarized in Table 5. According to the analysis, Infermedica was associated with the lowest proportion of patients requiring high-level resources, followed by the ESI, while the Manchester Triage System had the highest proportion. Patients in the Infermedica group were less likely to require high levels of ED resources compared to those in the Manchester Triage System group (OR 0.51, 95% CI, 0.45–0.58, *P* < .001). These results show differences in resource utilization among the triage methods, with Infermedica associated with a lower proportion of high-level use, while the ESI and Manchester Triage System tended to show a higher inclination toward high resource utilization.

Medical error rates across the three triage methods are summarized in Table 6. We defined medical errors as incorrect triage category assignments that resulted in delayed or inappropriate treatment. The structured chart reviews performed by two independent emergency physicians using Worster and Bledsoe methodology identified these errors. The inter-rater reliability reached a strong level according to the Cohen kappa coefficient of 0.81. The established framework enabled standardized and objective assessment of triage-related errors across different systems. The analysis indicates that the Infermedica triage method was associated with the lowest rate of medical errors, while higher error rates were observed in both the ESI and Manchester Triage System methods.

Table 6 presents medical error analysis. The medical error rates were determined through a structured chart review of 900 randomly selected cases (300 per triage group) rather than the entire cohort of 18,000 patients. According to the analysis, Infermedica demonstrated the lowest mortality rate, followed by ESI, while the Manchester Triage System had the highest mortality rate. Adjusted logistic regression analysis showed that the likelihood of mortality was significantly lower in the Infermedica group compared to the Manchester Triage System (OR 0.39, 95% CI, 0.32–0.47, *P* < .001) and ESI (OR 0.61, 95% CI, 0.51–0.74, *P* < .001).

Legal issue outcomes for the three triage methods are summarized in Table 7. The analysis shows that Infermedica was associated with the lowest rate of medicolegal issues, while the ESI and Manchester Triage System had higher rates. The odds of encountering legal issues were significantly lower in patients triaged with Infermedica than with the Manchester Triage System (OR 0.28, 95% CI, 0.20–0.40, *P* < .001) and the ESI (OR 0.42, 95% CI, 0.30–0.59, *P* < .001).The inter-rater reliability for medical error assessment was strong (Cohen kappa 0.81). These results indicate that Infermedica had the lowest rate of medicolegal issues, while the Manchester Triage System had the highest rate of legal issues.

## DISCUSSION

This study demonstrated that an AI-assisted triage system was associated with reduced patient wait times, and fewer medical errors and medicolegal issues compared to the ESI and Manchester Triage System; however, the observational design precludes firm conclusions about causality, with no definitive relationship established between triage processes and clinical outcomes, resource utilization, medical error rates, and legal issues.^15^,^16^ The findings show that each triage method had distinctly different impacts on ED dynamics, and especially highlight the significant advantages of the AI-assisted triage method in this context.

In this study, each triage cohort contained exactly 6,000 patients. This was achieved by using a computer-generated stratified randomization algorithm rather than relying on simple randomization. Patients were first identified from the pool of all eligible ED visits during the study period. The algorithm assigned patients to one of the three triage methods (Infermedica, ESI, the Manchester Triage System) in a balanced way while controlling for age and sex distributions to avoid baseline demographic imbalances. This stratified approach ensured equal sample sizes across cohorts, thereby improving the statistical power of comparisons and maintaining comparability of clinical and operational outcomes. Such stratified randomization is a standard and intentional design method when exact cohort sizes are needed and is not the result of random chance.

Infermedica was associated with shorter patient wait times compared to ESI and the Manchester Triage System. These findings should be interpreted cautiously as other operational factors could influence patient wait times.^17^,^18^ The observed reduction in wait time with the AI-assisted triage system primarily reflects its ability to automate and accelerate the *initial risk assessment and categorization* process. Because Infermedica integrates patient-reported symptoms, vital signs, and medical history in real time, it decreases the time nurses spend navigating manual flowcharts or subjective decision-making. Although our wait-time metric measures the interval up to first physician evaluation (not just the triage process itself), faster triage decisions likely improved overall patient flow, enabling physicians to see patients sooner. Additionally, standardized recommendations and reduced misclassification may have minimized the need for re-triage or reassessment, indirectly shortening the path to physician contact.

One potential explanation for the shorter wait times and lower number of error rates observed with the AI-assisted system is the automated data processing and risk stratification it performs. Unlike ESI and the Manchester Triage System, which rely on manual navigation of flowcharts and nurse judgment, the AI algorithm integrates patient-reported symptoms, vital signs, and medical history into a standardized risk score in real time. This reduces delays caused by subjective decision-making and the need to consult complex manual protocols. Additionally, AI-supported triage can process large amounts of clinical information simultaneously and present recommendations within seconds. Because these models are built on extensive medical knowledge bases and pattern recognition, they may reduce human error and misclassification, especially with atypical or overlapping symptoms.

In contrast, ESI and the Manchester Triage System depend heavily on nurse experience and can vary between users, while AI-assisted systems provide more consistent outputs. Despite its structured algorithms, an AI-driven triage system may produce inaccurate, or “hallucinated,” recommendations when faced with atypical or incomplete data. This phenomenon—known as algorithmic hallucination—has been documented in several AI-based clinical support systems and represents a key operational risk. Such errors may occur when models extrapolate beyond their training distributions, misinterpret rare symptom combinations, or produce overconfident recommendations without acknowledging uncertainty. Emergency departments implementing AI-assisted triage should, therefore, incorporate real-time monitoring tools, fail-safe alerts, and clinician override mechanisms to mitigate the impact of potentially unsafe outputs. Such errors may arise from limitations in the training dataset or model drift over time. These safeguards, including continuous monitoring, feedback loops, and clinician override capability, would enable clinicians to detect and correct unsafe outputs before they affect patient care. However, these potential advantages should be interpreted cautiously because staff enthusiasm, initial workflow adjustments, and the single-center design could have influenced performance differences.

The algorithm-based system helped decrease manual delays in triage, which led to shorter patient wait times. The outcome could have been influenced by other factors such as staff engagement with new technology and workflow optimizations. The algorithmic triage process led to shorter wait times because it performs assessments automatically without delays related to human judgment. The advantage of shorter wait times might have been influenced by confounding factors, which include the level of familiarity of triage nurses with each system, the complexity of patient symptoms, and the workflow adjustments made during the initial implementation phase.^19^,^20^ The models included adjustments for key confounding variables (eg, age, sex, vital signs, comorbidities), yet unmeasured factors such as staff enthusiasm for new technology or inherent system efficiency may still have contributed to observed differences. Infermedica’s ability to rapidly and accurately evaluate patients’ clinical conditions facilitated faster triage decisions and enhanced patient flow within the ED. In contrast, the longer wait times observed with ESI—and particularly with the Manchester Triage System—suggest that the manual and less time-sensitive structure of these methods may be insufficient in managing growing patient volumes. This inefficiency may negatively impact both patient satisfaction and clinical outcomes.^21^

In terms of resource utilization, Infermedica was found to allow for more balanced and efficient resource use. Resource utilization is a critical parameter for efficiency and cost effectiveness in the ED, and this study shows that the AI-assisted Infermedica triage method provided a more optimal distribution of resources. In contrast, the ESI and Manchester Triage System triage methods were associated with higher resource utilization rates. This suggests that traditional triage methods may require more resources, potentially reducing ED efficiency. These findings also suggest that AI-assisted triage systems could be advantageous in terms of cost effectiveness.

When analyzing medical error rates, it was observed that the AI-supported triage method had the lowest rate of medical errors. Although the observed medical error rates were lowest with Infermedica, even a seemingly small percentage of triage misclassification may have meaningful clinical consequences in a high-volume ED. In high-acuity emergency settings, even minor misclassification can translate into delayed recognition of life-threatening conditions, unnecessary resource allocation, or inappropriate patient flow. Small deviations in initial triage accuracy may produce cascading clinical effects, including prolonged treatment times, increased morbidity, and heightened operational strain. Therefore, understanding the clinical implications of error patterns is crucial for evaluating the real-world safety profile of AI-supported triage.

Incorrect urgency assignment can delay life-saving interventions, prolong patient suffering, and increase morbidity. Our analysis highlights that AI-based systems may help reduce this risk; however, error reduction does not mean elimination. A single mis-triaged unstable patient could lead to rapid deterioration and medicolegal exposure, underscoring the importance of continuous human oversight and ongoing validation of AI recommendations. Since medical errors can have serious impacts on patient safety and clinical outcomes, this finding highlights that AI-assisted triage systems may play a key role in enhancing patient safety.

The higher medical error rates of the ESI and Manchester Triage System triage methods could have been attributed to their reliance on manual evaluation processes and increased susceptibility to human error. These findings strongly suggest that AI-assisted systems can provide a safer triage process by minimizing the risk of errors. From a clinical standpoint, the relatively lower error rates associated with Infermedica suggest a potential reduction in downstream adverse events, particularly for patients whose conditions may deteriorate rapidly without timely intervention. Conversely, higher error rates in manual triage systems underscore the vulnerability of human-dependent decision pathways, especially in crowded and high-pressure conditions.

In terms of medicolegal issues, Infermedica was found to have the lowest rate of legal complications. While our findings show reduced legal events with AI-assisted triage, introducing algorithmic decision support also creates new questions. Furthermore, the legal frameworks governing AI-assisted clinical decisions remain underdeveloped in many healthcare systems. Questions regarding shared liability between clinicians, healthcare institutions, and commercial developers are unresolved. In cases where AI output contributes to an adverse outcome, determining the extent of clinician reliance vs algorithmic responsibility becomes complex. Clear legal guidelines, standardized auditing systems, and transparent documentation of AI recommendations are essential to reduce medicolegal ambiguity and ensure defensible clinical practice.

Determining liability in cases where AI advice contributes to patient harm remains legally complex, and current regulations may not fully address shared responsibility between clinicians and developers. Institutions deploying AI triage should ensure clear protocols for accountability, maintain audit trails of AI outputs, and provide clinicians with final decision-making authority. Legal issues are critically important for the quality and safety of healthcare services and represent a significant risk factor for both healthcare professionals and institutions. The low rate of legal issues in Infermedica suggests that AI-assisted triage systems may reduce medicolegal risks by making more consistent and accurate decisions. In contrast, the higher rates of legal issues with the ESI and Manchester Triage System triage methods indicate that these systems may be more prone to errors in patient assessments, leading to a greater likelihood of legal complications.^22^,^23^

These findings suggest that AI-powered triage systems may reduce legal risks and enhance patient safety. In this study, Infermedica was associated with more favorable outcomes across several domains, but these findings should be interpreted in light of potential biases and the single-center design. Additionally, these findings require additional interpretation because they seem unexpected. Several mechanisms could explain these findings. The AI-supported system delivered faster and more reliable patient data processing than manual triage approaches, which minimized delays and human mistakes. The new AI system implementation during its initial phase likely improved staff performance through better engagement and protocol compliance. The system’s standardized interface helped reduce the variability among triage nurses, which resulted in uniform decision-making. While this study used stratified randomization and confounder adjustment, potential biases and contextual elements might still have affected the results. The promising results need cautious interpretation. Additional multicenter studies are needed to validate their general applicability.

Our study’s findings reveal that the AI-assisted triage method offers significant advantages in patient triage within EDs. Several studies published in 2025 further support and contextualize our results.^24^ Those studies, which evaluated deep learning-based triage systems, multimodal ED risk-prediction models, and AI-enhanced decision support, demonstrated substantial improvements in patient flow, early deterioration detection, and clinical prioritization accuracy. These studies consistently highlight that AI models outperform manual triage in identifying high-risk patients and reducing time to evaluation, aligning closely with the patterns observed in our findings. However, they also emphasize the necessity of continuous model validation, transparent uncertainty quantification, and robust governance structures to prevent unsafe reliance on algorithmic outputs. Integrating these insights strengthens the scientific context of our study and underscores the relevance of our results within the rapidly evolving ED-AI literature.

New large-scale evaluations of AI-driven triage in emergency care reported consistent reductions in patient wait times and improved risk stratification; they also emphasized the need for robust clinical oversight to manage algorithmic uncertainty and ethical implications.^25^ Integrating these findings with our data suggests that AI-supported triage can enhance safety and efficiency but must be deployed along with clear governance frameworks, real-time performance auditing, and clinician education to maintain patient trust and minimize harm.^21^ The association of Infermedica with shorter wait times, balanced resource utilization, and lower rates of medical errors and legal issues suggests that this method could be a more effective and safer option for ED management. Although ESI and the Manchester Triage System still have a wide range of use, it is clear that these traditional methods have notable disadvantages when compared to AI-based systems.^26^

## LIMITATIONS

This study has several important limitations that should be considered when interpreting the findings. First, the research was conducted at a single, high-volume ED, which may limit the generalizability of the results to other institutions with different patient populations, resources, and operational structures. Second, the relatively short observation period could have introduced novelty bias, as staff may have been more engaged and motivated during the initial implementation of the AI-assisted system. Third, patient satisfaction was measured through self-reported surveys, which are subject to response bias and may not fully capture objective experience; additionally, differences in survey administration methods (face-to-face vs phone) were not formally controlled. Fourth, the assessment of medical errors was based on a structured review of a representative sample of cases rather than the entire cohort, which could have led to sampling bias despite high inter-rater reliability.

Moreover, although the statistical models adjusted for key confounding variables, residual and unmeasured confounders may still have existed and could have influenced the observed differences. Importantly, no formal a priori sample size or power calculation was performed, which limits the ability to determine whether the study was adequately powered to detect smaller but clinically meaningful differences. In addition, no correction for multiple comparisons was applied; therefore, *P*-values should be interpreted with caution due to the increased risk of type I error.

Finally, the study evaluated only short-term outcomes within a single-center setting. Long-term patient outcomes, system-wide cost impacts, and broader workflow effects were not assessed. Future multicenter studies with extended follow-up are needed to validate these findings and explore the generalizability and sustainability of AI-assisted triage performance across diverse healthcare environments.

## CONCLUSION

The integration of AI-assisted triage systems in the ED has the potential to improve patient safety, ensure resource efficiency, and elevate the overall quality of healthcare services.

## Figures and Tables

**Table 1:** Age and sex distribution of 18,000 patients in a study assessing the effectiveness of three triage methods in the emergency department.

Age group	Total patients	Female patients (n, %)	Male patients (n, %)
18–25	1,801	901 (50.03%)	900 (49.97%)
26–35	2,747	1,358 (49.45%)	1,389 (50.55%)
36–45	2,661	1,274 (47.89%)	1,387 (52.11%)
46–55	2,595	1,303 (50.21%)	1,292 (49.79%)
56–65	2,593	1,287 (49.64%)	1,306 (50.36%)
66–75	2,676	1,330 (49.71%)	1,346 (50.29%)
76–85	2,673	1,325 (49.56%)	1,348 (50.44%)

Stratified randomization was used to ensure demographic comparability between triage groups. Subgroup analyses confirmed no significant differences in age or sex distributions among the three triage groups (data not shown).

**Table 2:** Risk-management analysis by triage method.

Risk-management category	Infermedica (n, %)	ESI (n, %)	MTS (n, %)	P-value (ESI vs Infermedica)	P-value (MTS vs Infermedica)	Reference group
Good	3,557 (59.3%)	2,971 (49.5%)	2,436 (40.6%)	< .001	< .001	Infermedica
Moderate	1,850 (30.8%)	2,416 (40.3%)	2,366 (39.4%)	< .001	< .001	Infermedica
Poor	593 (9.9%)	613 (10.2%)	1,198 (20.0%)	.74	< .001	Infermedica

These results align with the goal of evaluating the safety and precision of triage methods.

*ESI*, Emergency Severity Index; *MTS*, Manchester Triage System.

**Table 3:** Patient satisfaction analysis by triage method.

Triage method	Number of patients	Average satisfaction score	Standard deviation	Minimum score	Maximum score	P-value (vs infermedica)
Infermedica	6,000	9.0	0.82	8	10	Reference
ESI	6,000	8.0	0.81	7	9	< .001
MTS	6,000	7.0	0.81	6	8	< .001

*ESI*, Emergency Severity Index; *MTS*, Manchester Triage System.

**Table 4:** Average patient wait times by triage method (mean ± SD).

Triage method	Average wait time (minutes)	Standard deviation (minutes)
Infermedica	14.95	5.02
ESI	25.06	7.04
MTS	34.94	10.08

*ESI*, Emergency Severity Index; *MTS*, Manchester Triage System.

**Table 5:** Resource utilization analysis by triage method in a study of system-level resource impacts depending on triage system in use.

Triage method	High resource utilization (%)	Moderate resource utilization (%)	Low resource utilization (%)	Total number of patients	P-value (High vs Infermedica)
Infermedica	29.9% (1,797)	49.8% (2,986)	20.3% (1,217)	6,000	Reference
ESI	41.4% (2,482)	39.3% (2,358)	19.3% (1,159)	6,000	< .001
MTS	48.7% (2,919)	30.4% (1,821)	21.0% (1,260)	6,000	< .001

*ESI*, Emergency Severity Index; *MTS*, Manchester Triage System.

**Table 6:** Medical error analysis by emergency department triage method (based on random sample of 300 patients per cohort).

Triage method	No medical error (%) (n)	Medical error present (%) (n)	Total number of patients	P-value (vs Infermedica)
Infermedica	99.07% (5,943)	0.93% (57)	6,000	Reference
ESI	97.83% (5,870)	2.17% (130)	6,000	< .001
MTS	97.30% (5,838)	2.70% (162)	6,000	< .001

*ESI*, Emergency Severity Index; *MTS*, Manchester Triage System.

**Table 7:** Incidence of medicolegal issues reported in association with each triage system.

Triage method	No legal issues (%) (n)	Medicolegal issue present (%) (n)	Total number of patients	P-value (vs Infermedica)
Infermedica	99.17% (5,950)	0.83% (50)	6,000	Reference
ESI	98.07% (5,883)	1.93% (116)	6,000	< 0.001
MTS	97.10% (5,826)	2.90% (174)	6,000	< 0.001

*ESI*, Emergency Severity Index; *MTS*, Manchester Triage System.
